# The Effect of Shyness on Adolescent Network Problem Behavior: The Role of Gender and Loneliness

**DOI:** 10.3389/fpsyg.2020.00803

**Published:** 2020-06-03

**Authors:** Peng Wang, Yun Yan, Fengqiang Gao, Ruifang Zhang, Jun Wang, Xiangping Zhan, Yu Tian

**Affiliations:** Department of Psychology, Shandong Normal University, Jinan, China

**Keywords:** shyness, loneliness, network problem behavior, gender, mediating moderation model

## Abstract

With the latest, rapid developments of the Internet, young people have become the main group in the online world. Congruently, Internet problem behaviors have shown a significant growth trend among adolescents. The present paper explores the factors affecting adolescents’ problem network behavior from the perspective of their shyness, gender, and loneliness, and provides suggestions for guiding these young people toward using the network rationally. The study surveyed 5,130 teenagers from Shandong province in China to investigate the moderating effect of gender on the relationship between shyness and problem network behavior, and the mediating effect of loneliness on the moderating effect. The results indicated that the level of shyness among girls was significantly higher than that among boys, whereas the prevalence of cyberbullying, pathological Internet use, and Internet gaming disorder was significantly lower for girls than for boys. The relationship among shyness, cyberbullying, and Internet gaming disorder was found to be moderated by gender, and the problems of cyberbullying and Internet gaming disorder faced by shy boys were greater than those faced by shy girls. In addition, the moderating effect of gender on cyberbullying and Internet gaming disorder was found to occur through the mediating factor of loneliness. The paper concludes with a discussion of the theoretical significance and generalizability of our research results.

## Introduction

Within the fast-paced development of modern society, Internet use has become an important activity in the daily lives of young people worldwide (Gómez et al., 2017). The 43rd Statistical Report on Internet Development in China confirmed that, as of December 2018, the proportion of the country’s students using the Internet in this way was 25.4%. The characteristics of the Internet mean that it offers continuous and significant opportunities for growth and improvement for teenagers. However, Internet use is like a double-edged sword: utilized reasonably (such as literature search, video call, and so on), it provides convenience and connectedness, whereas unreasonable network use (such as online games and so on) can cause young people to experience a number of network problem behaviors and may have a very serious negative impact on the physical and mental health of individuals.

### The Relationship Between Shyness and Network Problem Behavior

In the online environment, individuals generally abide by certain social rules. Yet, in this context, some people may demonstrate behaviors that deviate from these social norms, use the Internet unreasonably, or display hindered individual social adaptation.

In the present study, such “network problem behavior” specifically encompasses pathological Internet use (hereafter, “PIU”), Internet gaming disorder, and cyberbullying. PIU can be understood as an individual’s inability to control his or her use of the Internet, resulting in negative effects (Spada, 2014). Davis (2001) proposed a cognitive-behavioral model, which divided PIUs into special PIUs and general PIUs. Generally, PIU refers to the fact that there is no destination to rely on the network, and special PIU refers to the excessive use of the Internet by Internet users for specific purposes, such as the addiction of online games. In this study, PIU refers specifically to general PIU. Online gaming is a relatively new type of entertainment that combines traditional games with the Internet, thereby offering enhanced interactivity and virtuality aspects, but, for teenagers in particular, a condition referred to as Internet gaming disorder has been linked to a variety of behavioral problems (Zhang et al., 2016). Because of its major mental health impact, the loss of control over online gaming was termed “Internet gaming disorder” (IGD) (Ko, 2014). Cyberbullying refers to the intentional bullying of others by information technology through the Internet or mobile network, which is the behavior that the wider public strive to avoid (Furlong et al., 2004; Zhao and Gao, 2012). An example is making rude or mean comments on others on social networking sites. It is often accompanied by other negative behaviors or outcomes, such as poor peer relationships and low academic performance (Katzer et al., 2009), which may cause adolescents to experience a variety of physical and mental problems and have a negative impact on individual academic performance and healthy growth (Wanda, 2013).

Shyness is a personality trait encompassing a type of inhibition or discomfort shown by individuals in interpersonal situations that will significantly affect their participation in activities (Henderson and Zimbardo, 2001; Lo Coco et al., 2018). As a personality trait, shyness is a key factor affecting individual behavioral characteristics. Studies have found that individuals with shy tendencies account for 48% of the total sample (Heiser et al., 2003). Moreover, a study by Lei and Zhang (2002) found that shyness was a significant variable in predicting bullying. Shy individuals commonly demonstrate greater social avoidance, which may make it more difficult for other people to accept them and so they instead become bullies (Ren et al., 2018). At the same time, shy individuals may also show more aggression due to a hostile attribution tendency (Gao et al., 2016a, b).

In recent years, research has identified a relationship between shyness and network problem behavior. According to Young’s (1999) ACE model, the characteristics of online anonymity, convenience, and escapism could help shy individuals reduce discomfort in real interactions and could improve their social skills through social networking and build good relationship with others. However, if a shy individual relies too much on the compensation of the Internet without restraint, it may develop into PIU (Kraut et al., 1998). What’s more, according to reinforcement theory, the slot-machine mechanism or the ever-increasing use of technology is one of the elements of IGD, and the improvements in technology that facilitate this can make an individual feel psychologically satisfied, and thereby allow them to avoid or eliminate the discomfort brought about through real life. Shy people are more likely to escape from reality and achieve satisfaction in this way (Lei and Zhang, 2002). Furthermore, national and international scholars alike have observed a positive correlation between shyness and PIU, with some studies concluding that shyness is a typical personality trait of online game addicts (Zhang et al., 2006).

### The Mechanisms of Shyness and Network Problem Behavior

While the relationship between shyness and problem network behavior has been established, the moderating (e.g., gender) and mediating (e.g., loneliness) mechanisms between the two remain underexplored.

Loneliness is a negative emotional experience, and it occurs when the quality and quantity of social relationships an individual expects to have differ significantly from the actual situation. Related research shows that personality traits are related to individual loneliness. Loneliness is a negative consequence of shyness, and the two are significantly positively related (Aydin et al., 2013). Some studies have directly pointed out that shyness is a prerequisite for loneliness (Zambarano, 2001). Due to lack of social skills and social support, shy individuals are apt to create obstacles in their interaction with others and to avoid social evasion and rejection behaviors, which makes them more prone to loneliness. At the same time, studies have found that shy individuals are more likely to experience peer relationship difficulties and more likely to experience loneliness (Zhao et al., 2012). High shyness often coexists with a strong sense of loneliness. Shyness is an important predictor of loneliness. Lonely individuals are more inclined to use social networking to regulate negative emotions to compensate for their poor social skills in real life. A study that used adolescents as subjects showed that lone individuals would use the Internet more frequently to communicate private topics and meet new friends (Bonetti et al., 2010).

Several studies have found that loneliness triggers increased levels of online behavior (Hamburger and Ben-Artzi, 2003; Sahin, 2012; Feng et al., 2018). Indulging in the Internet for long periods of time may greatly reduce an individual’s communication with others in real life, and the exchange of genuine emotions may become less common. Lonely individuals are more inclined to use social networks to regulate their negative emotions, yet excessive Internet dependence may lead to an addiction to it (Ping et al., 2011). In addition, individuals who suffer from loneliness are more likely to be dissatisfied with their existing relationships and to perceive others’ neutral behaviors as being aggressive, which may cause cyberbullying (Olenikshemesh et al., 2012). The emotional deficiency of lonely individuals in real life may be resolved through online social activities and emotional communication during games played in the virtual world (Griffiths et al., 2004).

According to the social role theory, gender can lead to differences in social behaviors by influencing social role expectations (i.e., gender role expectations) and individual beliefs or skills (i.e., gender role performance) (Xie D. Z. et al., 2015). Both shyness and problem network behavior have been found to exhibit gender differences. Prior studies have shown that the relative levels of girls’ shyness are significantly higher than those of boys (Liu et al., 2012). Sherer (1997) first posited that men were more likely to be addicted to the Internet than women. Levels of boys’ Internet addiction have since been found to be significantly higher than those of girls as well (Zhang et al., 2011). Compared with girls, boys have more experience in playing online games (Zhang et al., 2014) and stronger motivation in respect of such activity. At the same time, the cyberbullying levels of boys are also significantly higher than those of girls (Shou and Chen, 2015). What’s more, some studies explored gender as a moderating variable and found that the relationships among shyness and loneliness/social avoidance, psychological safety, and Internet problem behavior were moderated by gender (Xie J. L. et al., 2015; Zhou and Liu, 2015; An, 2017).

### Hypothetical Model

At present, there is no prior research that directly examines the moderation effect of gender with respect to the relationship between shyness and problem network behavior and the mechanism behind it. The present study proposes to explore the relationship between adolescent shyness and online problem behaviors on the basis of gender differences, and to explore the mediation effect of loneliness.

In addition, studies have found that PIU groups are younger than those exhibiting addictive behaviors, with adolescents between the ages of 12 and 18 being most likely to develop PIU symptoms (Tsai and Lin, 2004). Accordingly, this study selected as its sample participants students from the sixth grade of primary school to college-age students in order to obtain comprehensive data pertaining to the investigation of the research question.

**FIGURE 1:**
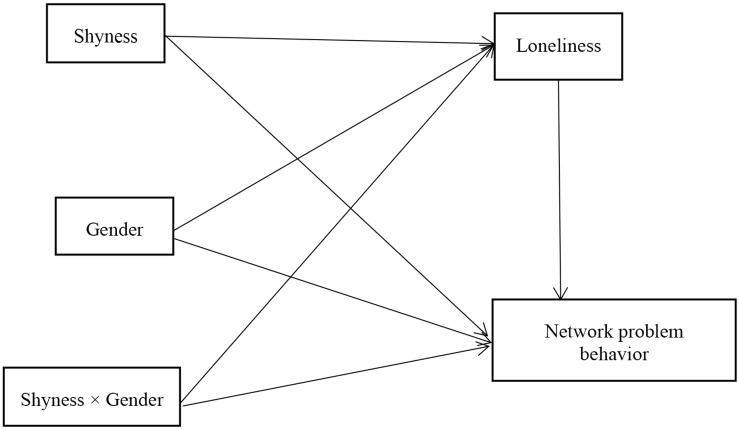
Hypothesis model of the relationship between shyness and problem network behavior.

Based on the aforementioned discussions, the following hypotheses and theoretical model (Figure 1) were proposed:

Hypothesis 1. The shyness level of girls is significantly higher than that of boys.

Hypothesis 2. Shyness is significantly positively related to PIU, IGD, and cyberbullying.

Hypothesis 3. The relationship between shyness and network problem behavior is moderated by gender, and the issue of shy boys is greater than that for girls.

Hypothesis 4. The moderating effect of gender on shyness and network problem behavior is realized through the mediating variable of loneliness.

## Materials and Methods

This study conformed with the code of ethics of the World Medical Association (Declaration of Helsinki) for experiments involving humans and was approved by the Ethics Committee of Shandong Normal University. Additionally, our research obtained written informed consent from the parents of the participants.

### Participants

Students from primary school (sixth grade class), junior high school, senior high school, and university all in the city were randomly selected in Eastern China. Participants were selected through a combination of stratified sampling and simple random sampling. A total of 5,500 questionnaires were distributed. After eliminating the invalid questionnaires (a scale with half or more of its contents unanswered was considered invalid), 5,130 valid questionnaires were returned, representing an effective response rate of 93.27%. Among these participants, 537 were primary school students (10.5%), 1,673 were junior high school students (32.6%), 675 were senior high school students, and 2,245 were university students (43.8%); 2,303 were boys (44.89%) and 2,827 were girls (55.11%), and their ages ranged from 10 to 23 years old (average age, 16.20 ± 3.24 years).

### Procedures

Taking the class as the unit, we carried out the group test in the classroom. All of the experimenters have a master’s degree in psychology. The head teachers of each class assisted in conducting the tests on the primary, middle, and high school student participants in order to manage and improve the quality of the completed questionnaires. The questionnaires were completed by and data collected from the students in the classroom.

### Measures

#### The Revised Henderson Undergraduate Shyness Scale

The Revised Henderson Undergraduate Shyness Scale (RHUSS) was compiled by Henderson and Zimbardo (2002) and the revised one by Wang et al. (2009) was used. The revised scale showed high reliability and validity in the Chinese youth sample study (Wang et al., 2009). The revised shyness scale consisted of 17 topics, divided into four dimensions, namely, seeking approval, self-blame, fear of rejection, and self-restriction of expression. One sample item is, “I worry about being immature in social situations.” It featured a five-point scale (1 = *strongly disagree*, 5 = *strongly agree*), and the higher the score, the higher the level of shyness. In this study, Cronbach’s alpha coefficient with respect to the scale was 0.869. We also conducted CFA, with *x*^2^*/df* = 14.408, *p* < 0.001, RMSEA = 0.047, CFI = 0.948, IFI = 0.949, AGFI = 0.956, and GFI = 0.970.

#### The UCLA Loneliness Scale

The UCLA loneliness scale compiled by Russell (1996) was translated and revised to obtain a Chinese version. One sample item is, “I am unhappy doing so many things alone.” The scale had a total of 20 questions and used four-point numerical response rating (1 = *never*, 4 = *always*). The higher the total score, the higher the level of loneliness. For the reverse measure, we carried out reverse scoring. In this study, Cronbach’s alpha was 0.855. We also conducted CFA, with *x*^2^*/df* = 10.934, *p* < 0.001, RMSEA = 0.044, CFI = 0.966, IFI = 0.967, AGFI = 0.956, and GFI = 0.975.

#### Cyberbullying Scale

The cyberbullying scale adapted from Ybarra et al. (2007) was translated and revised to obtain a Chinese version. Three questions were presented and participants responded to them according to a seven-point scale, with the numerical response ratings ranging from 1 (*strongly disagree*) to 7 (*strongly agree*). One sample item is, “Make rude or mean comments on others on social networking sites.” The higher the total score, the higher the online bullying level. In this study, Cronbach’s alpha coefficient with regard to the scale was.85. We also conducted CFA, with *x*^2^*/df* = 15.151, *p* < 0.001, RMSEA = 0.053, CFI = 0.984, IFI = 0.971, AGFI = 0.958, and GFI = 0.981.

#### Generalized Pathological Internet Use Scale

The Generalized Pathological Internet Use Scale (GPIUS) originally prepared by Gómez et al. (2017) was translated and revised, and a Chinese version of the scale was obtained. It had a total of 11 questions and used a seven-point rating scale (1 = *strongly disagree*, 7 = *strongly agree*). One sample item is, “You have connected to the Internet even though you knew it could get you in trouble.” The higher the total score, the higher the degree of PIU. In this study, Cronbach’s alpha was 0.883. We also conducted CFA, with *x*^2^*/df* = 15.996, *p* < 0.001, RMSEA = 0.054, CFI = 0.980, IFI = 0.980, AGFI = 0.963, and GFI = 0.982.

#### Internet Gaming Disorder Test (IGDT-10)

The IGD test compiled by Király et al. (2017) was translated and revised, and a Chinese version of the test was obtained. The scale consisted of 10 items, encompassing “continue,” “focus,” “negative consequences,” “escape,” “tolerance,” “lost control,” “abandonment of other activities,” “deception,” “avoidance,” and the measurement of individual IGD obstacles in nine dimensions. One sample item is, “When you were not playing, how often have you fantasized about gaming, thought of previous gaming sessions, and/or anticipated the next game?” It featured a seven-point numerical response scale, ranging from 1 (*strongly disagree*) to 7 (*strongly agree*); the higher the total score, the higher the use of online games. In this study, Cronbach’s alpha coefficient of the scale was 0.915. We also conducted CFA, with *x*^2^*/df* = 10.253, *p* < 0.001, RMSEA = 0.042, CFI = 0.996, IFI = 0.996, AGFI = 0.978, and GFI = 0.994.

### Statistical Analysis

We used SPSS (version 19.0) statistical analysis software to manage and analyze the data, mainly using statistical methods for describing statistics, the correlation analysis, and the analysis of variance. Confirmatory factor analysis was performed using the AMOS 24.0 software package. The mediation effect was analyzed using SPSS 19.0.

To test the common method biases, the common variance analysis was conducted by the factor analysis. Then we analyzed the statistics via descriptive and correlation analyses. In addition, we examined the mediating roles of gender and loneliness.

For moderating effect, when the product coefficient of the predictive variable and gender is significant, we consider the moderating effect to be significant. The bootstrapping method was conducted to test the mediation effects. This method produced 95% bias-corrected confidence intervals of these effects from 1,000 resamples of the data. Confidence intervals that did not contain zero indicated that effects were significant.

## Results

### Common Method Biased

Using self-reporting methods to collect data is likely to lead to common method bias, and so this study used Harman’s single factor analysis to test for it (Podsakoff et al., 2003). The unrotated principal component factor analysis showed that the characteristic root value of nine factors were greater than 1, and the variance explained by the first factor was only 20.005%, which is less than the critical standard of 40%, indicating that there was no obvious common method bias in the study.

### Descriptive Statistical Analysis

Results from the descriptive statistical analysis are shown in Table 1.

**TABLE 1:** Descriptive statistical analysis of all variables.

	**Primary school**		**Junior high school**		**Senior high school**		**University**	
	**Boy**	**Girl**	**Boy**	**Girl**	**Boy**	**Girl**	**Boy**	**Girl**
Shyness	45.503 (9.967)	45.696 (10.206)	44.578 (11.857)	45.300 (11.179)	46.850 (13.078)	48.848 (13.478)	45.879 (11.646)	47.642 (10.620)
Loneliness	41.076 (9.093)	41.224 (9.472)	41.487 (9.308)	41.550 (8.960)	43.156 (10.650)	42.000 (9.935)	42.155 (9.275)	41.316 (8.543)
Cyberbullying	4.472 (2.654)	4.301 (2.347)	5.133 (3.269)	4.612 (2.499)	5.216 (3.592)	4.429 (2.740)	5.620 (3.295)	4.057 (2.115)
PIU	30.611 (13.279)	26.217 (11.639)	38.199 (13.865)	35.268 (13.438)	39.170 (14.191)	40.072 (13.677)	38.354 (12.480)	39.391 (11.508)
Internet gaming disorder	24.194 (12.485)	18.220 (9.302)	30.121 (13.899)	21.847 (11.235)	28.198 (14.647)	20.277 (12.667)	29.452 (12.548)	20.365 (10.451)

Taking gender and study stage as independent variables, and shyness, loneliness, cyberbullying, PIU, and IGD as dependent variables, multivariate analysis of variance was conducted. The results showed that gender and study stage had significant interaction effects on loneliness, *F*_(__3_,_5122__)_ = 2.638, *p* < 0.05, η^2^ = 0.002; the interaction effect on PIU was significant, *F*_(__3_,_5122__)_ = 12.022, *p* < 0.001, η^2^ = 0.007; the interaction effect on cyberbullying is significant, *F*_(__3_,_5122__)_ = 15.657, *p* < 0.001, η^2^ = 0.009. The interaction between shyness and IGD was not significant. Further simple effect analysis showed that the level of loneliness had significant differences between boys and girls in the university group, and the level of loneliness in boys was significantly higher than that in girls (*p* < 0.001). There were significant differences between boys and girls in the primary and junior high school groups. The PIU level of boys was significantly higher than that of girls (*p* < 0.001); cyberbullying had significant gender differences in junior high school, senior high school, and college groups, and boys’ cyberbullying levels were significantly higher than those of girls (*p* < 0.001).

The main effect of gender in shyness was significant, *F*_(__1_,_5122__)_ = 9.471, *p* < 0.05, η^2^ = 0.002; the level of shyness of girls was significantly higher than that of boys; the main effect of gender in cyberbullying was significant, *F*_(__1_,_5122__)_ = 67.064, *p* < 0.001, η^2^ = 0.013; boys’ cyberbullying level was significantly higher than that of girls. What’s more, PIU’s gender main effect was significant, *F*_(__1_,_5122__)_ = 9.965, *p* < 0.05, η^2^ = 0.002; boys’ PIU level was significantly higher than that of girls. The main effect of gender in IGD was significant, *F*_(__1_,_5122__)_ = 380.621, *p* < 0.001, η^2^ = 0.069; the level of boys’ IGD was significantly higher than that of girls.

The main effect of study stage in shyness was significant, *F*_(__3_,_5122__)_ = 13.652, *p* < 0.001, η^2^ = 0.008. The level of shyness of senior high school students was significantly higher than that of junior high school students and primary school students. College students’ shyness level was significantly higher than that of junior high school students. The main effect of study stage in loneliness was significant. *F*_(__3_,_5122__)_ = 3.496, *p* < 0.05, η^2^ = 0.002. High school students’ loneliness level was significantly higher than that of primary school students. The main effect of study stage in cyberbullying was significant, *F*_(__3_,_5122__)_ = 4.440, *p* < 0.01, η^2^ = 0.003. Junior high school students’ cyberbullying level was significantly higher than that of primary school students. The main effect of study stage in PIU was significant, *F*_(__3_,_5122__)_ = 104.832, *p* < 0.001, η^2^ = 0.058. The PIU level of college students was significantly higher than primary school students and junior high school students. Senior high school students’ PIU level was significantly higher than that of primary school students and junior high school students. What’s more, junior high school students’ PIU level was significantly higher than that of primary school students. The main effect of study stage in IGD was significant, *F*_(__3_,_5122__)_ = 21.831, *p* < 0.001, η^2^ = 0.013. The IGD level of junior high school students was significantly higher than that of primary school students, high school students, and college students. High school students’ IGD level was significantly higher than that of primary school students, and college students’ IGD level was significantly higher than that of primary school students.

### Correlation Analysis

Results from the correlation analysis are shown in Table 2. Gender was not significantly related to PIU, but all other variables were significantly related to each other. There was a significant negative correlation between gender and loneliness/cyberbullying/IGD. In addition, other variables were positively correlated with each other.

**TABLE 2:** Correlation analysis of all variables.

		**1**	**2**	**3**	**4**	**5**	**6**
1	Gender	1	–	–	–	–	–
2	Shyness	0.061**	1	–	–	–	–
3	Loneliness	−0.038**	0.450**	1	–	–	–
4	Cyberbullying	−0.168**	0.126**	0.235**	–	–	–
5	PIU	−0.021	0.369**	0.282**	0.306**	1	–
6	Internet gaming disorder	−0.323**	0.213**	0.238**	0.415**	0.511**	1

**p < 0.01

### The Relationship Between Shyness and Network Problem Behavior: Mediating Moderation Model

Following Ye and Wen (2013), an intermediary moderation model test method was used to examine whether gender’s moderation of the relationship between shyness and problem network behavior plays a role in loneliness.

The first step was to establish a relationship model among shyness (*X*), gender (*U*), cyberbullying, PIU, and IGD (*Y*), and to test whether the relationship between shyness and network problem behavior is moderated by gender. To avoid multiple collinearity, the relevant variables were centered. The regression equation was as follows:

(1) $$Y=c_{0}+c_{1}\,X+c_{2}\,U+c_{3}\,U\,X+e_{1}$$

The results are shown in Table 2.

As can be seen from Table 3, the interaction between shyness and gender had a significant effect on cyberbullying and IGD, indicating that the direct effect of shyness on cyberbullying and IGD was regulated by gender. The effect of the interaction between shyness and gender on PIU was not significant, which suggests that the direct effect of shyness on PIU was not regulated by gender.

**TABLE 3:** Simple moderation model test results: relationship among shyness and cyberbullying, PIU, and Internet gaming disorder.

**Dependent variable**	**Independent variables**	**β**	***t***	***R*^2^**	***DR*^2^**	***F***
Cyberbullying	Shyness	0.033	9.681***	–	–	72.958***
	Gender	−1.021	−13.063***	0.046	–	–
	Shyness × Gender	−0.041	−6.109***	0.053	0.007	–
PIU	Shyness	0.417	28.177***	–	–	258.724***
	Gender	−1.490	−4.365***	0.168	–	–
	Shyness × Gender	−0.007	−0.222	0.168	0.000	–
Internet gaming disorder	Shyness	0.257	17.923***	–	–	251.260***
	Gender	−8.741	−26.445***	0.159	–	–
	Shyness × Gender	−0.154	−5.376***	0.163	0.004	–

***p < 0.001.

**FIGURE 2:**
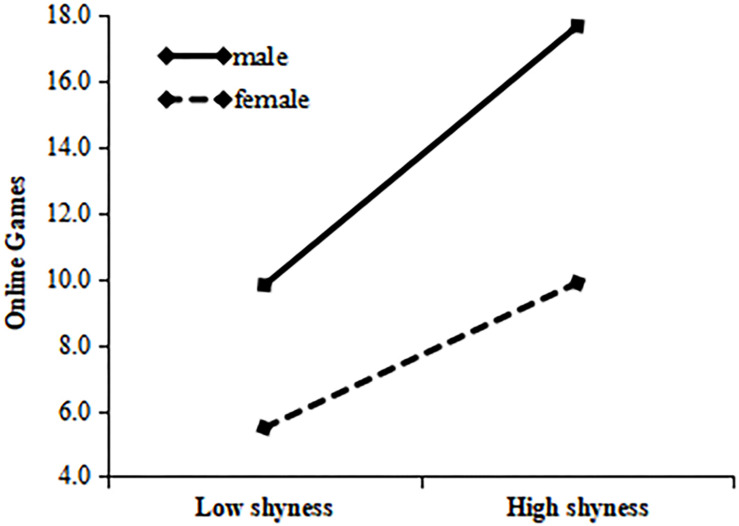
Gender’s role in moderating between shyness and cyberbullying.

A simple slope test showed that, for cyberbullying, the predictive coefficient β of shyness in the male population (i.e., boys) was 0.055, *p* < 0.001; in the female population (girls), the predictive coefficient β was 0.016, *p* < 0.001. This indicates that shyness has a significant relationship on cyberbullying, but the relationship is weaker with respect to girls than to boys. The specific results are illustrated in Figure 2.

**FIGURE 3:**
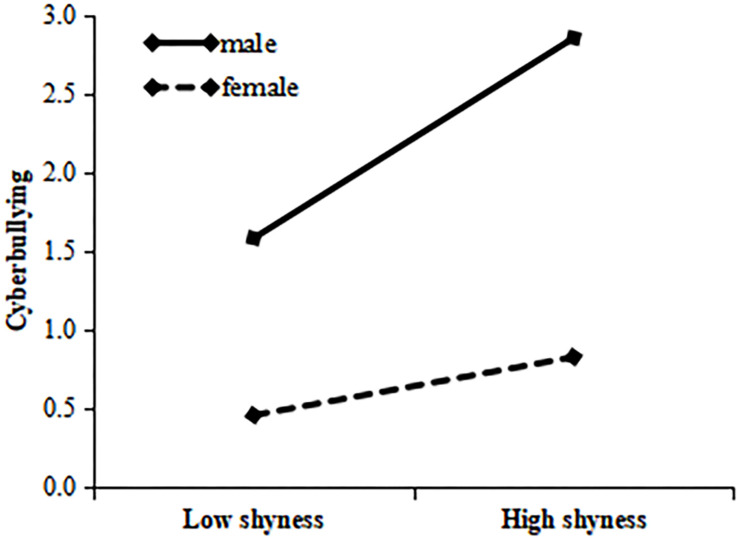
Gender’s role in moderating between shyness and Internet gaming disorder.

For IGD, in the boys’ group, shyness had significant relationship with IGD, with the prediction coefficient β being 0.34, *p* < 0.001; in the girls’ group, the coefficient was reduced to 0.19, *p* < 0.001. This indicates that shyness has a significant relationship with IGD, but the relationship is weaker with respect to girls than to boys. The specific results are shown in Figure 3.

The second step was to establish a relationship model among shyness (*X*), gender (*U*), loneliness (*W*), and their interactions with cyberbullying and IGD (*Y*), and to test the mediating role of loneliness in the moderation model expounded above. The regression equations were as follows:

(2) $$W=a_{0}+a_{1}\,X+a_{2}\,U+a_{3}\,U\,X+e_{2}$$

(3) $$Y=c_{0}^{\prime }+c_{1}^{\prime }\,X+c_{2}^{\prime }\,U+c_{3}^{\prime }\,U\,X+b_{1}\,W+b_{2}\,U\,W+e_{3}$$

The results are shown in Table 4.

**TABLE 4:** Mediating moderation model test results: relationship among shyness and cyberbullying and Internet gaming disorder.

**Dependent variable**	**Independent variables**	**β**	***t***	***R*^2^**	***DR*^2^**	***F***
Loneliness	Shyness	0.360	36.416***	–	–	335.275***
	Gender	−1.192	−5.213***	0.207	–	–
	Shyness × Gender	0.027	1.367	0.207	0.000	–
Cyberbullying	Shyness	0.009	2.515*	–	–	85.992***
	Gender	−0.938	−12.215***	0.046	–	–
	Shyness × Gender	−0.032	−4.285***	0.053	0.007	–
	Loneliness	0.066	13.998***	0.080	0.027	–
	Loneliness × Gender	−0.031	−3.327*	0.091	0.001	–
Internet gaming disorder	Shyness	0.179	11.286***	–	–	190.732***
	Gender	−8.481	−25.873***	0.159	–	–
	Shyness × Gender	−0.149	−4.689***	0.163	0.004	–
	Loneliness	0.215	10.733***	0.182	0.019	–
	Loneliness × Gender	−0.030	−0.745	0.182	0.000	–

*p < 0.05; ***p < 0.001.

As can be seen from Table 3, the effect of shyness on loneliness was significant, β = 0.360, *p* < 0.001; and the effect of gender and loneliness on cyberbullying was significant, β = −0.031, *p* < 0.05, which suggests that gender regulates the effect of loneliness on cyberbullying by indirectly regulating the effect of shyness on cyberbullying. The effect of gender and shyness on cyberbullying *c*’_3_ was significant, β = −0.032, *p* < 0.001, indicating that there were some mediations in the moderation effect. In the relationship between shyness and cyberbullying, the gender moderation effect *c*_3_ was −0.041, wherein the direct effect *c*’_3_ was −0.032, the indirect moderation effect *c*_3_–*c*’_3_ was −0.009, and the indirect moderation effect was 21.95%.

The effect of shyness on loneliness was found to be significant, β = 0.360, *p* < 0.001; the effect of gender and loneliness on the IGD was not significant, β = −0.030, *p* > 0.05; *b*_1_ was not significant, and *a*_3_ was not significant. Bootstrap interval tests were performed on *a*_3_*b*_1_, *a*_3_*b*_2_, and *a*_1_*b*_2_, where the confidence interval at 95% of *a*_3_*b*_1_ did not contain 0, indicating that the mediating effect of loneliness was significant; the effect of gender and shyness interaction on IGD *c*’_3_ was significant, β = −0.149, *p* < 0.001, indicating that the regulatory effect was partially mediated. Regarding the relationship between shyness and IGD, the gender moderation effect c_3_ was −0.154, wherein the direct effect *c*’_3_ was −0.149, the indirect moderation effect *c*_3_–*c*’_3_ was −0.005, and the indirect moderation effect was 3.25%.

## Discussion

This study examined the relationship between adolescent shyness and network problem behavior, the role of gender in moderating these relationships, and its mechanism of action. With reference to the relationship between shyness, IGD, and cyberbullying, a mediating moderation model was established. That is to say, the role of gender in the moderation was largely via the mediating factor of loneliness. The research results offer a particular practical benefit for developing understanding of the relationship between shyness and problem network behavior in relation to gender, and toward guiding young people to use online networks judiciously.

### The Impact of Shyness on Network Problem Behavior

Through correlation analysis, our research on the relationship between shyness and problem network behavior found that shyness and loneliness, cyberbullying, PIU, and IGD were significantly positively correlated, and, in the model in which shyness directly affected network problem behavior, shyness could significantly positively predict network problem behavior, which was consistent with previous research (Lei and Zhang, 2002).

Studies have found that shyness may have various adverse consequences, including effects on individual emotions and self-awareness (Henderson and Zimbardo, 2001). The anonymity of online networks and non-face-to-face communication methods can promote individual self-disclosure to some extent (McKenna et al., 2010). However, the Internet is also considered to be a communication method through which participants can risk alienating each other (Gómez et al., 2017). Individuals who are accustomed to online communication may further reduce opportunities for face-to-face communication in real life. Reducing adolescents’ ability to feel and participate in real-life situations may increase their levels of shyness. Yang and Tung (2007) found that individuals with shyness traits were more likely to have issues with Internet addiction than individuals without such traits. When the Internet becomes the main social tool used by shy individuals, they are more likely to form network dependencies (Lei and Zhang, 2002).

There are three models that can be used to explain IGD in shy individuals. One is the use and satisfaction theory. Suler (1999) found that the desire for social interaction was one of the needs of Internet addicts. When the total social needs in the real life of a shy individual are not met, they will turn to an online network; when the satisfaction that the network brings to the individual becomes stronger than that derived from real life, the shy individual may develop an Internet addiction. The second model pertains to the social-cognitive theory proposed by Bandura (1999), which emphasizes behavior, environment, and personal interaction. Shy individuals may have a negative perception of the environment and of others, and Internet addiction can result from this cognition. The third is the cognitive behavior model, as proposed by Davis (2001) from the perspective of psychiatry, through which it may be posited that the negative influence of the network on the shy individual is mainly caused by the negative cognition of the individual, which is unrelated to the nature of the Internet itself.

In addition, studies have shown that shy individuals typically behave highly aggressively (Gao et al., 2016a, b). According to the shy social adaptation model, shy individuals transform their self-blame caused by retreating and avoiding behavior into resentment against others, and then exhibit aggressive behavior. The present study confirmed that shyness was positively related to cyberbullying, and thus provided further evidence for the shy social adaptation model.

### The Role of Gender in Moderation of Shyness and Network Problem Behavior

The results of this study showed that there was a gender difference in the relationships among shyness, cyberbullying, and IGD. Specifically, boys were found to be more sensitive than girls, which is consistent with previous research (Zhang et al., 2011). The reason for this result may be found in social culture considerations, wherein boys and girls experience different social role expectations. Such social categorizations are typically more tolerant of women’s expression of negative emotions, and women are often told that they need to be protected; therefore, arguably, they are more motivated to seek help (Shou and Chen, 2015). Girls who seek help may reduce the negative effects of shyness by confiding in others to gain support, and thereby reduce network behavior problems. For boys, however, the social role expectations are that they will be stronger and braver; hence, when shy boys’ behaviors are inconsistent with male gender stereotypes, negative evaluations from peers may be triggered. At the same time, boys have been found to be more skilled and interested in online network operations (Zhang et al., 2014) and are more inclined to use the Internet to vent their negative emotions.

Equally, according to evolutionary psychology theory, the “adaptation problem” is a major challenge to be solved in the evolutionary process (Shang and Xiong, 2007). In the present study, girls’ cyberbullying and manifestations of IGD were found to be less problematic, and one of the possible reasons for this could be that girls are at a disadvantage in resource competition (Lian et al., 2017), which will motivate them to develop stronger environmental adaptability than boys to enhance their social adaptation function. Therefore, girls will be more adaptable in the face of a bad social environment caused by shyness, the negative impact of shyness for them will be smaller, and the possibility of it causing network problem behavior is also reduced.

Based on these inferences, when it comes to preventing IGD and cyberbullying, it would seem to be necessary to give shy boys more attention than shy girls. The effect of gender on the relationship between shyness and PIU was not found to be significant, which also suggests that we need to classify network problem behavior in a more detailed manner when we study the gender differences in the relationship between shyness and network behavior problems.

### The Role of Loneliness in Act of Shyness and Network Problem Behavior

This study found that loneliness plays a mediating role among shyness, cyberbullying, and IGD. Shyness can significantly predict loneliness and problem network behavior, and loneliness is significantly positively correlated with network problem behavior (Hamburger and Ben-Artzi, 2003; Sahin, 2012; Feng et al., 2018). Overall, our results suggest that shy adolescents are more likely to be lonely, which, in turn, increases the propensity for cyberbullying and IGD. This study identified the internal mechanism of shyness affecting network problem behavior; that is, individual loneliness due to shyness was the proximal factor of shyness affecting adolescent cyberbullying and IGD.

The positive correlation between loneliness and IGD may be explained by what is known as the “poor-to-rich” model, derived from the theory of social compensation, which postulates that the network can enhance the connection between individuals and others. The social deficits of lonely individuals in their real lives can be addressed through the interactivity of IGD and by gaining supportive interpersonal relationships (Valkenburg et al., 2005). According to the theory of compensation, lonely individuals will make up for interpersonal interactions that cannot be realized in real life through online networks. At the same time, lonely individuals usually lack social support and social skills and are unable to get help when they encounter problems. They are typically emotionally unstable and easily perceive others’ behaviors as bullying behaviors, which may lead to more cyberbullying behaviors.

## Implications

In order to reduce the occurrence of network problem behaviors and to help teenagers use the Internet reasonably, we put forward the following suggestions. First, parents and educators should guide young people to use online networks correctly, to prudently face all kinds of information and temptations therein, and to understand the pros and cons of the Internet. Second, educators should develop their own abilities accordingly, as well as be able to effectively identify shy young people, especially shy boys, and guide them toward adopting appropriate methods to alleviate the discomfort caused by shyness. In addition, parents and schools should guide young people to participate in additional outdoor activities, provide more offline communication platforms, and offer further opportunities for youth interpersonal interactions (Formica et al., 2017; Mannino and Faraci, 2017). Finally, different education measures should be taken for boys and girls to conduct earlier and more timely interventions for shy individuals in order to help them develop good online habits.

## Limitations and Future Directions

The study had limitations, as follows. First, the cross-sectional research method used herein may have its own disadvantages, and so the relationship between the related variables requires further longitudinal study to validate and support our findings. Second, this study focused on the mediating role of loneliness, but future research could investigate whether other negative emotions such as anxiety and depression have similar mediating effects. Despite these limitations, however, this research provides further empirical evidence that shyness and loneliness should be considered as personal traits that are relevant to network problem behavior, as well as documenting that there are different effects in adolescents according to their gender. However, future study can explore the relationship between multidimensional social competence and cyberbullying, and face-to-face harassment and cyberbullying (García-Fernández et al., 2015; Romera et al., 2017). In addition, there may be different impact mechanisms on the PIU problem of disabled and homosexual people in the subject group, which needs more attention (Mannino and Schiera, 2017; Mannino et al., 2017). Finally, the present research was conducted in a Chinese cultural setting, and the cross-cultural applicability of the conclusions must be properly verified. Related research should be conducted in different countries and cultures.

## Conclusion

(a) The shyness level of girls was significantly higher than that of boys, and the gender difference of cyberbullying, PIU, and IGD was significantly lower than that of boys.

(b) Shyness was positively correlated with loneliness, cyberbullying, PIU, and IGD.

(c) The relationship among shyness, cyberbullying, and IGD was moderated by gender; the issue of shy boys is greater than that of girls.

(d) The moderating effect of gender on cyberbullying and IGD was achieved through the mediating factor of loneliness, which suggested that shyness had a gender difference in the effects of cyberbullying and IGD, and loneliness played a mediating role.

## Data Availability Statement

The datasets generated for this study will not be made publicly available. The datasets for this manuscript are not publicly available because the datasets are used only for the team of this article by the permission of the guardians. Requests to access the datasets should be directed to PW, 122394108@qq.com.

## Ethics Statement

This study conformed with the code of ethics of the World Medical Association (Declaration of Helsinki) for experiments involving humans and was approved by the Ethics Committee of Shandong Normal University. Additionally, our research obtained written informed consent from the parents of the participants.

## Author Contributions

PW is the research designer. YY is in charge of writing. FG and YT participated in the discussion and offered suggestions. RZ is the first corresponding author. JW is the second corresponding author. XZ is the third corresponding author.

## Conflict of Interest

The authors declare that the research was conducted in the absence of any commercial or financial relationships that could be construed as a potential conflict of interest.
